# Association of social worker-assessed psychosocial factors with 30-day hospital readmissions among hemodialysis patients

**DOI:** 10.1186/s12882-018-1162-4

**Published:** 2018-12-17

**Authors:** Olufunmilola Adisa, Bernard G. Jaar, Tahsin Masud, Abyalew Sahlie, Catherine Obadina, Joshua Ang, Janice P. Lea, Laura C. Plantinga

**Affiliations:** 1 Department of Epidemiology, Rollins School of Public Health, Emory University, Atlanta, GA USA; 2 Department of Medicine, Johns Hopkins School of Medicine, Baltimore, MD USA; 3 Welch Center for Prevention, Epidemiology and Clinical Research, Johns Hopkins University, Baltimore, MD USA; 4 Department of Epidemiology, Johns Hopkins Bloomberg School of Public Health, Baltimore, MD USA; 5 Nephrology Center of Maryland, Baltimore, MD USA; 6 Division of Renal Medicine, Department of Medicine, Emory University, Atlanta, GA USA; 7 Philadelphia College of Osteopathic Medicine, Suwanee, GA USA

**Keywords:** Hemodialysis, Hospital readmissions, Psychosocial factors, Mental health, Social worker

## Abstract

**Background:**

Evidence regarding the effect of psychosocial factors on hospital readmission in the setting of hemodialysis is limited. We examined whether social worker-assessed factors were associated with 30-day readmission among prevalent hemodialysis patients.

**Methods:**

Data on 14 factors were extracted from the first available psychosocial assessment performed by social workers at three metropolitan Atlanta dialysis centers. Index admissions (first admission preceded by ≥30 days without a previous hospital discharge) were identified in the period 2/1/10–12/31/14, using linked national administrative hospitalization data. Readmission was defined as any admission within 30 days after index discharge. Associations of each of the psychosocial factors with readmission were assessed using multivariable logistic regression with adjustment for patient and index admission characteristics.

**Results:**

Among 719 patients with index admissions, 22.1% were readmitted within 30 days. No psychosocial factors were statistically significantly associated with readmission risk. However, history of substance abuse vs. none was associated with a 29% higher risk of 30-day readmission [OR: 1.29, 95% CI: 0.75–2.23], whereas depression/anxiety was associated with 20% lower risk [OR: 0.80, 95% CI: 0.47–1.36]. Patients who were never married and those who were divorced, or widowed had 38 and 17% higher risk of 30-day readmission, respectively, than those who were married [OR: 1.38, 95% CI: 0.84–2.72; OR: 1.17, 95% CI: 0.73–1.90].

**Conclusions:**

Results suggest that psychosocial issues may be associated with risk of 30-day readmission among dialysis patients. Despite the limitations of lack of generalizability and potential misclassification due to patient self-report of psychosocial factors to social workers, further study is warranted to determine whether addressing these factors through targeted interventions could potentially reduce readmissions among hemodialysis patients.

## Background

In 2016, the Centers for Medicare & Medicaid Services (CMS) spent $28 billion on hemodialysis in the United States; about one-third of end-stage renal disease (ESRD) expenditures were for inpatient care [1]. As part of its ESRD Quality Incentive Program, CMS ties reimbursement of U.S. ESRD services to clinical performance [2]. In 2017, this pay-for-performance program, added the standardized readmission ratio (SRR), such that facilities’ Five-Star ratings may be reduced due to higher-than-expected hospital readmissions among their hemodialysis patient population. One of the major criticisms of the SRR is that, while it accounts for some demographic and clinical factors, it does not account for differences in psychosocial factors across facilities, which may disadvantage facilities with disproportionately vulnerable populations [3].

Despite this, relatively little is known about the effect of psychosocial factors on readmissions among hemodialysis patients. In a recent single-center study, El-Majzoub et al. [4] found that psychosocial distress was associated with shorter time to hospitalization, but did not examine hospital readmissions specifically. Flythe et al. [5] found that poor social support and depressive symptoms were associated with higher risk of hospital readmissions among dialysis patients in a prospective study. Both studies used validated tools that were administered in a study setting. However, it is possible that data collected routinely by social workers as part of usual hemodialysis care could capture a wide variety of psychosocial factors and potentially inform providers of patient risk of subsequent hospital readmission without the need for additional assessments. Thus, we aimed to use clinically available psychosocial information addressing a variety of domains, extracted from both structured and unstructured electronic medical record (EMR) data from three metropolitan Atlanta dialysis centers, to identify social worker-assessed psychosocial factors associated with risk of 30-day readmission among dialysis patients.

## Methods

### Study design and population

**Fig. 1:**
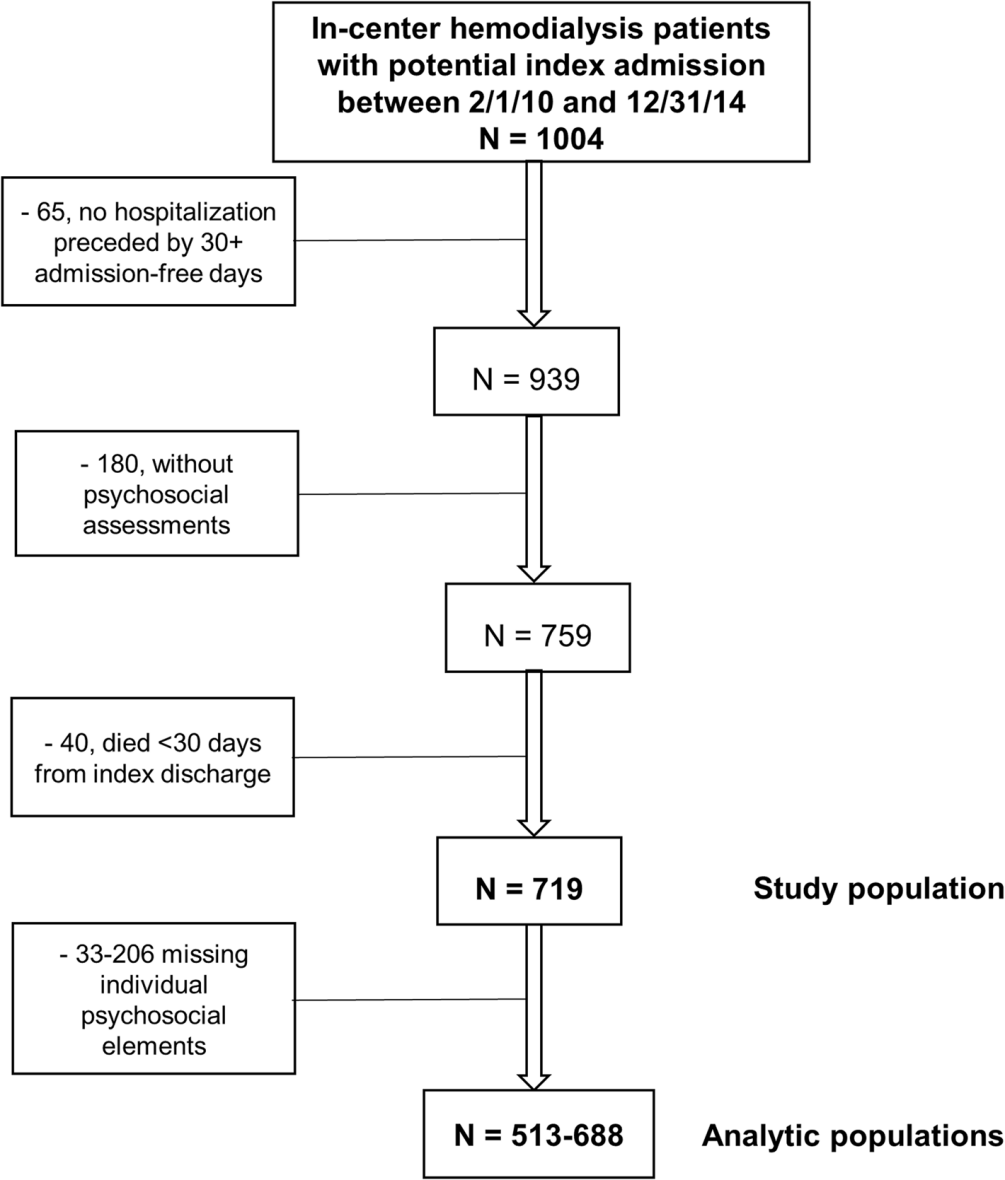
Selection of patient population

Data for this study were obtained from the EMR used by the three clinics operated by Emory Dialysis and from linked United States Renal Data System (USRDS) data [1]. The study was approved (with waiver of patient consent) by the Emory Institutional Review Board. We identified 1004 index hospitalizations in the period from 2/1/10 to 12/31/14, using the linked USRDS hospitalization file. Patients were excluded if they had no hospitalizations preceded by ≥30 days with a previous discharge (*n* = 65), if they did not have a baseline social worker assessment (*n* = 180), or died < 30 days from the index discharge (*n* = 40), leaving a study population of 719 index admissions (Fig. 1). For psychosocial variables, we extracted data from the first available social worker assessment for each patient. For analysis of individual psychosocial factors, index admissions were further excluded for missing data for that factor (*n* = 33–206), resulting analytic population sizes of 513–688 (Fig. 1).

### Study variables

#### Readmission

Readmission was defined as any admission within 30 days after discharge from the index admission. Index admission was defined as the first admission that was preceded by ≥30 days without a previous hospital discharge, to mitigate influence of frequently readmitted patients. Admission information was obtained from linked hospitalization data. Pulmonary edema-related readmissions were defined via discharge diagnoses International Classification of Diseases, Ninth Revision (ICD-9) codes of fluid overload (276.6, 276.61, or 276.69), heart failure (428.x, 402.× 1, 404.× 1, 404.× 3, or 398.91), or pulmonary edema (518.4 or 514), in any position [6, 7].

#### Psychosocial factors

Assessments were performed using a form common to all social workers in all three facilities. The assessment items associated with the variables reported here can be found in Table 1. Psychosocial factors were categorized into four domains:

**Table 1:** Items from social worker assessments used to define psychosocial factors

Psychosocial factors	Question/item	Possible responses
Mental health domain		
History of substance abuse	Has the patient ever had a history of substance abuse?	• No • Yes
Depression or anxiety	Are there signs/symptoms present for depression or anxiety problems?	• No • Yes
Social support domain		
Relationship status	What is the patient’s relationship status?	• Domestic partner • Married • Divorced • Single • Widowed • Separated
Living alone	With whom does the patient live?	• Lives alone • Lives with parents • Spouse • Children • Significant other • Friend • Relative • Other
Frequency of social support	What is the level of involvement of family and friend on a regular basis?	• Daily • Weekly • Monthly • Less frequently than monthly
Independence domain		
Impaired memory	Does the patient appear to have a problem with the following?	Short term memory • No • Yes Long term memory • No • Yes
Current employment	Current employment?	• Employed full-time • Employed part-time • Retired • Medical leave of absence • Unemployed-by choice • Unemployed disabled • Unemployed-looking for work • Other
Mobility impairment	Ambulatory assistance?	• None • Cane/crutch • Walker or • Manual wheelchair • Electric wheelchair • Limb prosthesis
Type of housing	Living status?	• Home • Condo • Mobile home • Apartment • Rents house • Assisted living • Homeless • Public housing shelter • Long term care facility(SNF) • Acute rehabilitation center • Correctional facility • Adult family home • Adult group home
Ease of adherence domain		
Coming to dialysis	Over the past month, how easy or difficult has it been for you to come to each hemodialysis treatment?	• N/A • Very easy • Somewhat easy • Neither easy nor difficult • Somewhat difficult • Very difficult
Completing dialysis sessions	Over the past month, how easy or difficult has it been for you to complete the full-prescribed hemodialysis treatment time?	• N/A • Very easy • Somewhat easy • Neither easy nor difficult • Somewhat difficult • Very difficult
Taking medications	Over the past month, how easy or difficult has it been for you to take medications as prescribed?	• N/A • Very easy • Somewhat easy • Neither easy nor difficult • Somewhat difficult • Very difficult
Adhering to dietary restrictions	Over the past month, how easy or difficult has it been for you to follow dietary restrictions?	• N/A • Very easy • Somewhat easy • Neither easy nor difficult • Somewhat difficult • Very difficult
Adhering to fluid restrictions	Over the past month, how easy or difficult has it been for you to follow fluid restrictions?	• N/A • Very easy • Somewhat easy • Neither easy nor difficult • Somewhat difficult • Very difficult

*N/A* Not applicable

##### Mental health domain

The history of substance abuse was defined by patient report and information about substance abuse from prior medical information accessible to the social worker. Depression or anxiety was defined as social worker-assessed presence of symptoms or signs of depression or anxiety at the time of assessment.

##### Social support domain

Marital status was defined by the patient report, and categorized as domestic partner/married, never married, and divorced/widowed/separated. Living alone was defined as not living with any other individual, including parents, children, other family, or friends. Frequency of social support from family and/or friends was dichotomized as daily vs. less than daily.

##### Independence domain

Memory status was defined based on the social worker’s observation of long-term or short-term memory impairment, which were combined as any vs. no memory impairment. Current employment was categorized as employed, disabled, and unemployed. The type of housing was dichotomized as community-dwelling vs. assisted living/nursing home. Mobility impairment was defined as “yes” to patient using an assistive device vs. none.

##### Ease of adherence domain

Patient ease with coming to dialysis sessions, completing dialysis sessions, taking medications, adhering to dietary restrictions, and adhering to fluid restrictions were reported to the social worker. Responses were dichotomized as difficult (“somewhat difficult” or “very difficult,” or “neither easy nor difficult”) vs. easy (“somewhat easy” or “very easy”).

#### Other variables

Patient age and duration of ESRD at index admission, sex, race, and index admission characteristics [length of stay and intensive care utilization (≥1 day in an intensive care or coronary care unit during the index admission)] were obtained from USRDS. Comorbid conditions were assigned if they appeared on the CMS-2728 Medical Evidence form or were present in discharge codes from all hospital discharges in the year up to and including the index admission, using the diagnostic codes outlined in the CMS Chronic Conditions Warehouse algorithms [8]. The assigned cause of ESRD and insurance type were recorded at the start of dialysis on the CMS-2728.

### Statistical analysis

Patient and index admission characteristics were summarized. The burden of readmissions was determined as the percentage of index admissions that resulted in a readmission within 30 days of discharge from the index admission. Associations of each of the psychosocial factors with readmission [odds ratios (ORs)] were assessed using multivariable logistic regression analyses with and without adjustment for potential confounders. Additional adjustment for insurance type and for time between psychosocial assessment and index admission was performed in sensitivity analyses. In secondary analyses examining whether psychosocial factors might differentially affect readmissions for pulmonary edema (fluid overload) vs. other causes, we used multinomial logistic regression models to estimate adjusted ORs. SAS v. 10.4 (Cary, NC) and Stata v. 14.2 (College Station, TX) were used for analysis.

## Results

### Patient characteristics

The mean age of our predominantly black (91.8%) study population was 56; more than half (52.6%) were male (Table 2). Comorbid conditions were common, particularly hypertension, diabetes, congestive heart failure, and atherosclerotic cardiovascular disease. The median length of stay for the index admission was 4 days, and 21.7% were admitted to the ICU during the index admission. The median time between psychosocial assessment and index admission was 171 days. Overall, 22.1% of index admissions were followed by a 30-day readmission. Index admissions followed by a readmission vs. not were longer, but there were no other statistically significant differences in index admission or patient characteristics by readmission status (Table 2).

**Table 2:** Characteristics of prevalent metropolitan Atlanta hemodialysis patients with index admissions from 2010 to 2014

Characteristic	*N*	Overall	Readmitted (*n* = 159)	Not readmitted (*n* = 560)	P*
Patient characteristics					
Mean age (SD), years	*719*	56.1 (14.9)	55.2 (15.4)	56.3 (14.7)	*0.4*
Male sex, *n* (%)	*719*	378 (52.6)	78 (49.1)	300 (53.6)	*0.3*
Black race, *n* (%)	*718*	659 (91.8)	147 (92.5)	512 (91.6)	*> 0.9*
Medical insurance at dialysis start, *n* (%)	*692*				*0.5*
Medicare		122 (17.6)	34 (22.2)	88 (16.3)	
Medicaid		165 (23.8)	36 (23.5)	129 (23.9)	
Private		170 (24.6)	34 (22.2)	136 (25.2)	
Other		63 (9.1)	14 (9.2)	49 (9.1)	
None		172 (24.9)	35 (22.9)	137 (25.4)	
Comorbid conditions, *n* (%)	*719*				
Congestive heart failure		244 (33.4)	64 (40.3)	180 (32.1)	*0.06*
Hypertension		701 (97.5)	155 (97.5)	546 (97.5)	*> 0.9*
Diabetes		349 (48.5)	79 (49.7)	270 (48.2)	*0.7*
Atherosclerotic cardiovascular disease		141 (19.6)	34 (21.4)	107 (19.1)	*0.5*
Cause of ESRD, *n* (%)	*712*				*0.3*
Hypertension		308 (43.3)	49 (30.8)	144 (26.0)	
Diabetes		193 (27.1)	69 (43.4)	239 (43.2)	
Glomerulonephritis		73 (10.3)	18 (11.3)	55 (10.0)	
Other		138 (19.4)	23 (14.5)	115 (20.8)	
Median duration of ESRD (IQR), years	*719*	2.1 (0.6–5.9)	1.5 (0.5–5.4)	2.2 (0.7–6.3)	*0.1*
Index admission characteristics					
Median length of stay (IQR), days	*719*	4 (2–6)	4 (2–8)	3 (2–6)	*0.007*
Intensive care utilization, *n* (%)	*719*	156 (21.7)	42 (26.4)	114 (20.4)	*0.1*

Characteristics are assessed at index admission unless otherwise noted. *ESRD* End-stage renal disease, *IQR* Interquartile range, *SD* Standard deviation

*By chi-square, *t*, or rank-sum test, as appropriate

### Distributions of psychosocial factors

Table 3 shows the distribution of psychosocial factors by domain. In general, low levels of substance abuse and depression/anxiety and high levels of social support were reported. While many required ambulatory assistance, most were reported to be fairly independent. Most patients reported ease of adherence to coming to and completing dialysis sessions, taking medications, and adhering to dietary and fluid restrictions. There were no statistically significant differences in distributions of these factors by readmitted status (Table 3).

**Table 3:** Distributions of social worker-assessed baseline psychosocial factors among hemodialysis patients, by domain

Psychosocial domain/factor	*N*	*n* (%)			P*
		Overall	Readmitted	Not readmitted	
Mental health					
History of substance abuse, yes vs. no	*680*	90 (13.2)	22 (15.0)	68 (12.8)	*0.3*
Depression or anxiety, yes vs.no	*666*	114 (17.1)	21 (14.5)	93 (17.9)	*0.5*
Social support					
Marital Status	*686*				*0.3*
Never married		239 (34.8)	40 (26.5)	173 (32.3)	
Married/ domestic partner		213 (31.1)	59 (39.1)	180 (33.6)	
Divorced/separated/widowed		234 (34.1)	52 (34.4)	182 (34.0)	
Living alone	*688*	150 (21.8)	34 (22.7)	116 (21.6)	*0.8*
Daily social support	*673*	469 (69.7)	100 (67.6)	369 (70.3)	*0.5*
Independence					
Impaired memory	*678*	106 (15.6)	19 (13.1)	87 (16.3)	*0.3*
Employment at assessment	*542*				*0.4*
Employed		43 (7.9)	---**	37 (8.7)	
Disabled		314 (57.9)	67 (57.8)	247 (58.0)	
Unemployed		185 (34.1)	43 (37.1)	142 (33.3)	
Community-dwelling	*678*	623 (91.9)	135 (91.2)	488 (92.1)	*0.7*
Requires ambulatory assistance	*683*	278 (40.7)	55 (37.4)	223 (41.6)	*0.4*
Ease of adherence					
Easy to come to dialysis	*517*	397 (76.8)	92 (80.7)	305 (75.7)	*0.3*
Easy to complete dialysis	*513*	387 (75.4)	85 (75.9)	302 (75.3)	*0.9*
Easy to take medications	*527*	441 (83.7)	98 (83.1)	343 (83.9)	*0.8*
Easy to adhere to dietary restrictions	*523*	332 (63.5)	69 (60.0)	263 (64.5)	*0.3*
Easy to adhere to fluid restrictions	*519*	358 (69.0)	75 (65.2)	283 (70.1)	*0.3*

*By chi-square test. **Suppressed due to insufficient sample size (*n* < 10)

### Association of Psychosocial Factors with 30-day readmissions

No associations between psychosocial factors and 30-day readmissions were statistically significant, regardless of adjustment. However, Table 4 shows that some of the psychosocial factors examined were non-statistically significantly associated with 30-day readmission risk. For example, a history of substance abuse vs. none was non-statistically significantly associated with a 29% increased risk of 30-day readmission, whereas depression/anxiety at the time of assessment was non-statistically significantly associated with 20% lower readmission risk. For those who were never married and those who were divorced, separated, or widowed, vs. married, risks of 30-day readmission were 38 and 17% higher, respectively, but associations were non-statistically significant. Other factors reflecting social support, including living alone and less frequent social support, had null associations with readmission risk (Table 3). Memory impairment, being disabled vs. unemployed, and using assistive devices for ambulation were non-statistically significantly associated with 24, 20, and 25% lower readmission risk, respectively. Those who reported difficulty with coming to dialysis had 25% lower risk of 30-day readmission, whereas reported difficulties with adhering to dietary and fluid recommendations were associated with 20 and 22% higher risk, respectively, compared to reported ease of adherence, but associations were again non-statistically significant. Additional adjustment for insurance type at dialysis start and for time between psychosocial assessment and index admission did not change results substantially (data not shown).

**Table 4:** Association of social worker-assessed psychosocial factors with hospital readmissions among prevalent hemodialysis patients, 2010–2014

Psychosocial domain/factor	No. (%) readmitted within 30 days	OR (95% CI) for readmission	
		Unadjusted	Adjusted*
Mental health			
History of substance abuse			
Overall	147/680 (21.6%)	–	–
No	125/590 (21.2%)	1.00 (referent)	1.00 (referent)
Yes	22/90 (24.4%)	1.20 (0.72–2.02)	1.29 (0.75–2.23)
Depression or anxiety			
Overall	145/666 (21.8%)	–	–
No	124/552 (22.5%)	1.00 (referent)	1.00 (referent)
Yes	21/114 (18.4%)	0.78 (0.47–1.30)	0.80 (0.47–1.36)
Social support			
Marital status			
Overall	151/686 (22.0%)	–	–
Married/domestic partner	40/213 (18.8%)	1.00 (referent)	1.00 (referent)
Never married	59/239 (24.7%)	1.42 (0.90–2.23)	1.38 (0.84–2.27)
Widowed/divorced/separated	52/234 (22.2%)	1.24 (0.79–1.96)	1.17 (0.73–1.90)
Lives alone			
Overall	150/688 (21.8%)	–	–
No	116/538 (21.6%)	1.00 (referent)	1.00 (referent)
Yes	34/150 (22.7%)	1.07 (0.69–1.65)	1.16 (0.74–1.81)
Frequency of social support			
Overall	148/673 (22.0%)	–	–
Daily	100/469 (21.3%)	1.00 (referent)	1.00 (referent)
Not daily	48/204 (23.5%)	1.14 (0.77–1.68)	1.14 (0.76–1.70)
Independence			
Impaired memory			
Overall	145/678 (21.4%)	–	–
No	126/572 (22.0%)	1.00 (referent)	1.00 (referent)
Yes	19/106 (17.9%)	0.77 (0.45–1.32)	0.76 (0.44–1.32)
Employment status			
Overall	116/542 (21.4%)	–	–
Unemployed	43/185 (23.2%)	1.00 (referent)	1.00 (referent)
Disabled	67/314 (21.3%)	0.90 (0.58–1.38)	0.80 (0.49–1.32)
Employed	6/43 (14.0%)	0.54 (0.21–1.35)	0.54 (0.20–1.45)
Type of Housing			
Overall	148/678 (21.8%)	–	–
Community-dwelling	135/623 (21.7%)	1.00 (referent)	1.00 (referent)
Assisted living/nursing home	13/55 (23.6%)	1.12 (0.58–2.14)	1.07 (0.52–2.17)
Ambulatory assistance			
Overall	147/683 (21.8%)	–	–
No	92/405 (22.7%)	1.00 (referent)	1.00 (referent)
Yes	55/278 (19.8%)	0.84 (0.58–1.22)	0.75 (0.48–1.16)
Ease of adherence			
Coming to dialysis			
Overall	114/517 (22.1%)	–	–
Easy	92/397 (23.2%)	1.00 (referent)	1.00 (referent)
Difficult	22/120 (18.3%)	0.74 (0.44–1.25)	0.75 (0.44–1.27)
Completing dialysis			
Overall	112/513 (21.8%)	–	–
Easy	85/387 (22.0%)	1.00 (referent)	1.00 (referent)
Difficult	27/126 (21.4%)	0.97 (0.59–1.58)	0.98 (0.59–1.62)
Taking medications			
Overall	118/527 (22.4%)	–	–
Easy	98/441 (22.2%)	1.00 (referent)	1.00 (referent)
Difficult	20/86 (23.3%)	1.06 (0.61–1.84)	1.04 (0.59–1.82)
Adhering to diet restrictions			
Overall	115/523 (22.0%)	–	–
Easy	69/332 (20.8%)	1.00 (referent)	1.00 (referent)
Difficult	46/191 (24.1%)	1.21 (0.79–1.85)	1.20 (0.78–1.85)
Adhering to fluid restrictions			
Overall	115/519 (22.2%)	–	–
Easy	75/358 (21.0%)	1.00 (referent)	1.00 (referent)
Difficult	40/161 (24.8%)	1.25 (0.80–1.93)	1.22 (0.78–1.92)

*Adjusted for age, sex, race, duration of ESRD, history of diabetes, congestive heart failure, and atherosclerotic diseases, index admission length of stay, and intensive care utilization during index admission

In secondary analyses (Table 5), there were differences in the associations of pulmonary edema-related (31.5%) and other (68.5%) vs. no readmissions and several psychosocial factors. For example, living in assisted living or a nursing home vs. in the community was associated with 2.5-fold higher risk of pulmonary edema-related vs. no readmission, but 40% lower risk of other vs. no readmission. Difficulty adhering to fluid restrictions was associated with 67% higher risk of pulmonary edema-related readmissions only, and history of substance abuse was associated with 30% higher risk of other vs. no readmission only. Associations between the outcome and depression/anxiety, marital status, and frequency of social support, were similar for the two types of readmission vs. no readmission (Table 5).

**Table 5:** Association of social worker-assessed psychosocial factors with pulmonary edema-related and other hospital readmissions, vs. no readmissions, among prevalent hemodialysis patients, 2010–2014

Psychosocial domain/factor	Unadjusted OR (95% CI)	
	Pulmonary edema-related readmission vs. no readmission	Other readmission vs. no readmission
Mental health		
History of substance abuse		
No	1.00 (referent)	1.00 (referent)
Yes	1.00 (0.41–2.45)	1.30 (0.72–2.35)
Depression or anxiety		
No	1.00 (referent)	1.00 (referent)
Yes	0.67 (0.28–1.63)	0.83 (0.46–1.51)
Social support		
Marital status		
Married/domestic partner	1.00 (referent)	1.00 (referent)
Never married	1.36 (0.63–2.93)	1.44 (0.86–2.43)
Widowed/divorced/separated	1.35 (0.62–2.90)	1.19 (0.69–2.04)
Lives alone		
No	1.00 (referent)	1.00 (referent)
Yes	0.65 (0.28–1.50)	1.28 (0.79–2.07)
Frequency of social support		
Overall	–	–
Daily	1.00 (referent)	1.00 (referent)
Not daily	1.11 (0.58–2.11)	1.15 (0.73–1.81)
Independence		
Impaired memory		
No	1.00 (referent)	1.00 (referent)
Yes	1.05 (0.48–2.33)	0.65 (0.33–1.26)
Employment status		
Overall	–	–
Unemployed	1.00 (referent)	1.00 (referent)
Disabled	0.27 (0.03–2.15)	0.66 (0.24–1.83)
Employed	0.90 (0.45–1.82)	0.89 (0.54–1.49)
Type of Housing		
Community-dwelling	1.00 (referent)	1.00 (referent)
Assisted living/nursing home	2.45 (1.07–5.58)	0.60 (0.23–1.55)
Ambulatory assistance		
No	1.00 (referent)	1.00 (referent)
Yes	1.23 (0.67–2.26)	0.70 (0.45–1.10)
Ease of adherence		
Coming to dialysis		
Easy	1.00 (referent)	1.00 (referent)
Difficult	1.00 (0.46–2.19)	0.63 (0.33–1.20)
Completing dialysis		
Easy	1.00 (referent)	1.00 (referent)
Difficult	1.34 (0.64–2.83)	0.81 (0.45–1.48)
Taking medications		
Easy	1.00 (referent)	1.00 (referent)
Difficult	1.17 (0.50–2.78)	1.01 (0.53–1.93)
Adhering to diet restrictions		
Easy	1.00 (referent)	1.00 (referent)
Difficult	1.30 (0.65–2.59)	1.17 (0.71–1.92)
Adhering to fluid restrictions		
Easy	1.00 (referent)	1.00 (referent)
Difficult	1.67 (0.83–3.35)	1.08 (0.64–1.82)

## Discussion

We found that 22.1% of index admissions were followed by a readmission, similar to recent national studies using similar methodology [9]. Importantly, in this study examining social worker assessment-derived psychosocial factors and 30-day readmission risk among prevalent hemodialysis patients, associations were not statistically significant. However, our results do suggest that history of substance abuse, being unmarried, and patient-reported difficulty adhering to dietary and fluid restrictions may be associated with higher readmission risk, independent of patient and index admission factors. In contrast, depression/anxiety, impaired memory, requiring ambulatory assistance, being work-disabled or employed vs. unemployed, and patient-reported difficulty coming to dialysis were associated with lower readmission risk in our analyses. The other factors we examined—living alone, less frequent social support, community-dwelling vs. assisted living/nursing home, reported difficulty with completing dialysis sessions and taking medications—were not associated with readmission risk overall. However, analyses with a stratified outcome (pulmonary edema-related and other readmissions vs. no readmissions) suggested that living in assisted living or a nursing home and difficulty adhering to fluid restrictions or completing dialysis were associated with increased risk of pulmonary edema-related readmissions, while living alone was associated with increased risk of other readmissions.

Unexpectedly, we found that signs and symptoms of depression/anxiety at start of treatment were associated with 20% lower readmission risk, in contrast to the > 2-fold higher risk of hospitalization [4] and readmission [5] associated with positive screening for depression reported among hemodialysis patients, and to the 1.7-fold higher risk of readmission seen in hospitalized patients generally [10]. It is possible that patients thought by social workers to have depression/anxiety are followed more closely, resulting in lower readmission risk, or that these symptomatic patients are less likely to seek treatment, delaying readmissions past the 30-day threshold. However, this result could be partially attributable to differences in the timing of measurement between our study and these prior studies, if symptoms at discharge have more effect on readmission than chronic depression/anxiety. Social workers may also underdiagnosing depression and anxiety—which seems likely, given that about half of prevalent hemodialysis patients show signs of depression [11] and we found that only 17% of our population were noted to have depression at baseline. Since depression is associated with lower treatment adherence among dialysis patients [12, 13] and transplant recipients [13, 14], it may be important to assess depressive symptoms in this population more accurately and more frequently over time to determine readmission risk. Such work could lead to clinical interventions to reduce depressive symptoms including psychotherapy [15] and mindfulness meditation [16], which may effective in this population.

Non-married status was associated with higher risk of readmission, with no associations seen for frequency of social support or living alone. These results suggest that marital status may provide some protection beyond the presence of social support. However, it is also possible our measure of social support, which did not distinguish types [4] or providers of social support received, may not fully capture social support. Interestingly, several factors that indicate potential lack of independence in these hemodialysis patients, including impaired memory, requiring ambulatory assistance, and being work-disabled vs. unemployed were all associated with lower risk of readmission in our study. It is possible that such patients are followed more closely, both by dialysis providers and by caregivers, thus reducing the risk of readmission. Living in a nursing home or in assisted living, where patients are theoretically followed more closely than community-dwelling patients, did not provide this protection, but this discrepancy may be partially due to increased medical complexity of patients who are no longer community-dwelling. Furthermore, stratified outcomes suggested that, while those in assisted living/nursing homes were at lower risk of other readmissions, they were at higher risk of pulmonary edema-related readmissions. This may reflect a general protective effect of continuing medical care post-discharge, but poor post-discharge dialysis management among these patients. Because nursing homes are also now held accountable for readmissions [17], it may be important to explore better continuity of dialysis care specifically between nursing homes and dialysis facilities.

Difficulty with adherence was associated with both higher (dietary and fluid restrictions) and lower (coming to dialysis) risk of readmission. The prior association may reflect patient’s assessment of self-management skills, which are needed to understand and execute discharge instructions, including medication management—although we found no association with reported difficulty taking medications and readmission risk. The latter may reflect provider knowledge of and attempts to circumvent barriers to coming to dialysis (e.g., lack of transportation) among these patients. However, reported difficulty completing dialysis was not associated with overall readmission risk. These inconsistencies may reflect different types of adherence in dialysis being associated with different factors: for example, depression has been associated with missed dialysis [18] and medication adherence [13], but financial difficulties and provider and health system factors are also associated with problems taking medications [19]. Of course, these somewhat unexpected patterns could also be partially attributable to the misclassification due to self-reported data: while patients may report adherence to be “easy,” they may not find it to be easy in practice; furthermore, patients may not adhere to treatment recommendations for many reasons, related to trust, health literacy, polypharmacy, side effects, and financial barriers [20]. Culturally sensitive interventions to increase self-efficacy, individualized to patients’ problems with adherence, may be needed and may help prevent some readmissions [18, 21, 22].

There are several possible explanations for the lack of statistical significance in our results, including that psychosocial risk factors may not be associated with readmissions in the hemodialysis population. It is also possible that psychosocial factors do not contribute substantially to the ability of other demographic and clinical factors to predict readmissions. Flythe et al. [5] found that the associations between poor social support and depressive symptoms and higher risk of hospital readmissions among dialysis patients were independent of patient and index admission factors, making the explanation of a true null association less likely. Furthermore, in the general population, both patients [23] and providers [24] identify psychosocial variables as the primary reasons for readmissions, and it seems unlikely that these factors have no effect in the hemodialysis population. A future prospective study using validated instruments that are administered by social workers but standardized across multiple centers, or a retrospective study of a much larger dialysis organization (assuming standardized data collection by social workers), is needed to confirm these associations.

Other potential explanations for lack of statistical significance include inadequate statistical power, particularly in smaller subgroups; lack of an effect among our relatively homogeneous patient population, which is entirely urban and predominantly poor; potential selection bias due to missing data; and potential misclassification of the psychosocial factors. The potential for social desirability bias may be even stronger at the baseline assessment, since trust and rapport between social worker and hemodialysis patient may take time to establish [25]. Related to the timing of psychosocial assessment, misclassification is also possible due to the variable time lag between social worker assessment and index admission, which, on average, was around 6 months but was much longer for some patients. However, our results adjusting for this lag gave similar results.

Despite such limitations, our results do provide hypotheses that could be tested in future studies that inform clinical care and policy. Prior studies in the dialysis population have primarily focused on clinical risk factors for readmission [26–28], but the policy-driven nature of hemodialysis care has generated interest in other patient factors, including depression, social support, and health literacy [5], that could be modified to reduce readmission risk, or used for adjustment to make between-facility comparisons such as the dialysis SRR more fair. In our study, we leveraged existing social worker assessment data, which would be more readily available to policymakers, to examine the effects of multiple psychosocial factors addressing mental health, social support, independence, and ease of adherence on hospital readmission among hemodialysis patients.

There are additional limitations to this study that not already noted above. We excluded those patients with a recent previous hospitalization and those who died within 30 days from index discharge, which could lead to an underestimation of our outcome. Predictors of multiple readmissions or readmissions followed by death may differ from the predictors of the single readmission outcome examined here. While all social workers used the same form, there are no national standards for clinical collection of psychosocial information on patients and the assessment items on these clinical forms, including those for depression assessment, are not validated despite the availability of instruments for this population. Detailed information on medications used for depression/anxiety and on psychotherapy history was limited. Similarly, information on substance abuse did not include detailed information on duration or type of substance (e.g., intravenous vs. by mouth). However, strengths of the study include comprehensive evaluation of multiple psychosocial factors and linkage of data to administrative data with nearly complete capture of hospitalizations.

## Conclusions

This study adds to the body of knowledge on how psychosocial factors may affect 30-day readmission in hemodialysis patients. Our results could generate several hypotheses regarding psychosocial factors as potential predictors of readmission, potentially leading to future studies that inform polices and strategies to reduce readmissions among patients on hemodialysis. More frequent and robust collection of data on psychosocial factors, whether by the dialysis social worker or others, is needed. Such data may help guide targeted interventions to reduce readmissions, ultimately reducing costs and improving quality of life among hemodialysis patients.

## Abbreviations

CMS: Centers for Medicare & Medicaid Services
EMR: Electronic medical record
ESRD: End-stage renal disease
OR: Odds ratio
SRR: Standardized readmissions ratio
USRDS: United States Renal Data System
